# Pathogenic Mutations and Putative Phenotype-Affecting Variants in Polish Myofibrillar Myopathy Patients

**DOI:** 10.3390/jcm10050914

**Published:** 2021-02-26

**Authors:** Anna Potulska-Chromik, Maria Jędrzejowska, Monika Gos, Edyta Rosiak, Biruta Kierdaszuk, Aleksandra Maruszak, Andrzej Opuchlik, Cezary Zekanowski, Jakub P. Fichna

**Affiliations:** 1 Department of Neurology, Medical University of Warsaw, 1a Banacha St., 02-097 Warsaw, Poland; apotulska@wum.edu.pl (A.P.-C.); bkierdaszuk@wum.edu.pl (B.K.); aopuchlik@wum.edu.pl (A.O.); 2 Neuromuscular Unit, Mossakowski Medical Research Institute, Polish Academy of Sciences, 5 Pawinskiego St., 02-106 Warsaw, Poland; mjedrzejowska@imdik.pan.pl; 3 Department of Medical Genetics, Institute of Mother and Child, 17a Kasprzaka St, 01-211 Warsaw, Poland; monika.gos@imid.med.pl; 4 II Department of Radiology, Medical University of Warsaw, 1a Banacha St., 02-097 Warsaw, Poland; edyta.rosiak@yahoo.com; 5 Department of Neurodegenerative Disorders, Mossakowski Medical Research Institute, Polish Academy of Sciences, 5 Pawinskiego St., 02-106 Warsaw, Poland; aleksandra.maruszak@gmail.com (A.M.); c.zekanowski@imdik.pan.pl (C.Z.)

**Keywords:** skeletal muscle, NGS, WGS, genomic data, oligogenic inheritance, variant load

## Abstract

Myofibrillar myopathies (MFM) are heterogeneous hereditary muscle diseases with characteristic myopathological features of Z-disk dissolution and aggregates of its degradation products. The onset and progression of the disease are variable, with an elusive genetic background, and around half of the cases lacking molecular diagnosis. Here, we attempted to establish possible genetic foundations of MFM by performing whole exome sequencing (WES) in eleven unrelated families of 13 patients clinically diagnosed as MFM spectrum. A filtering strategy aimed at identification of variants related to the disease was used and included integrative analysis of WES data and human phenotype ontology (HPO) terms, analysis of muscle-expressed genes, and analysis of the disease-associated interactome. Genetic diagnosis was possible in eight out of eleven cases. Putative causative mutations were found in the *DES* (two cases), *CRYAB*, *TPM3*, and *SELENON* (four cases) genes, the latter typically presenting with a rigid spine syndrome. Moreover, a variety of additional, possibly phenotype-affecting variants were found. These findings indicate a markedly heterogeneous genetic background of MFM and show the usefulness of next generation sequencing in the identification of disease-associated mutations. Finally, we discuss the emerging concept of variant load as the basis of phenotypic heterogeneity.

## 1. Introduction

Myofibrillar myopathy (MFM) is a heterogeneous group of hereditary diseases of the skeletal muscles characterized by pathological myofibrillar disintegration and abnormal protein deposits. The myofibrils dissolution is centered around the Z-disc and is described as Z-disc streaming. This leads to ectopic aggregation of degraded granulofilamentous material in diverse patterns in the myofibril-free regions in proximity of the nuclei and sarcolemma. The accumulated proteins include but are not limited to those encoded by genes most commonly associated with the disease. Autophagic vacuoles containing degraded fragments of membranous organelles are also observed [1].

MFM should be diagnosed based on clinical presentation, electromyography, and especially on muscle biopsy findings [2], however, genetic results are a coveted confirmation of the diagnosis, and identification of the MFM type requires uncovering of the causative mutation.

The genetic basis of MFM is only partially understood. The majority of genetically diagnosed patients harbor pathogenic mutations in one of six genes: *DES*, *CRYAB*, *MYOT*, *LDB3*, *FLNC*, or *BAG3*. In addition, mutations in genes typically associated with other muscular diseases can result in MFM-like phenotypes. The list of such genes includes *FHL*, *TTN*, *DNAJB6*, *PLEC*, *ACTA1*, *HSPB8*, *LMNA*, *KY*, *PYROXD1*, *SQSTM1,* and *TIA1* and is still expanding [3]. Mutations in the *SELENON* gene are found among other disease entities in desmin-related myopathy with Mallory body-like inclusions (small clusters of accumulated proteins in the muscle fibers). *SELENON*-related muscle disorders are characterized by axial muscle weakness, spine stiffness, scoliosis, and serious breathing problems (https://ghr.nlm.nih.gov/gene/SELENON#conditions (accessed on 18 October 2020)).

Since many clinically MFM-like cases are left without genetic diagnosis, further efforts to uncover a genetic basis of the disease are justified. Next generation sequencing (NGS) followed by bioinformatic analyses of the obtained lists of genomic variants provide a wide, potentially non-biased approach towards discovering variants involved in pathogenesis [4]. This approach can identify novel mutations in known causative genes or even in genes not previously known to be related to a specific phenotype. Mutations in genes previously linked with other disorders may explain the observed clinical overlap between diverse muscle diseases. In addition to the causative genotype, some of the detected variants could also be phenotype-affecting, and some of them might even be deemed as co-causative.

Here, we report a whole exome sequencing study on a group of Polish patients with clinically diagnosed MFM. We identified pathogenic mutations in 73% of cases. These were found in genes already associated with myopathies, but not always typically with MFM. In some cases, we suggest additional, possible phenotype-affecting variants.

## 2. Materials and Methods

### 2.1. Patient Recruitment

The study involved thirteen patients from eleven unrelated families with clinically diagnosed myofibrillar myopathy from a single neuromuscular diagnosis and treatment medical center (see Table 1). The mean age of the patients at onset was 14 years (range 0–46), with nine patients exhibiting their first symptoms in childhood (0–9 years), two patients with the disease onset in early adulthood (21 and 26 years), and two patients with the disease onset in the fourth to fifth decade of life (39 and 46 years).

### 2.2. Ethics Approval and Consent to Participate

The study was approved by the Ethics Committee of the Warsaw Medical University and MSW Hospital (Warsaw, Poland) in compliance with national legislation and the Code of Ethical Principles for Medical Research Involving Human Subjects of the World Medical Association. Written consent was obtained from all study subjects according to the Declaration of Helsinki (BMJ 1991; 302:1194). The authors are very grateful to all for their participation in this study. 

### 2.3. Clinical Assessment

The patients were initially diagnosed, studied, and documented in the years 2003–2019 at the Department of Neurology, Medical University of Warsaw. The following were carried out for each patient: pedigree analysis; neurological exam including muscle strength assessment according to the Medical Research Council’s (MRC) grading scale; muscle biopsy; serum creatine kinase (CK) level determination; electrophysiological studies including nerve conduction tests and concentric needle EMG and, when indicated, cardiological examination with electrocardiography (EKG) and echocardiography; and respiratory function tests. In selected cases, an MRI scan of affected muscles was performed.

Genetic and clinical findings without reference to whole exome data have been published for patients 1a, 1b, 2, and 3 [5,6].

### 2.4. Genetic Analyses 

DNA was extracted from venous blood using standard methods [7]. Genomic DNA was captured using a SureSelect Human All Exon v5+UTR or SureSelect Human All Exon 6 (pts 7 and 8a) enrichment kit (Agilent Technologies, Santa Clara, CA, USA) and paired-end 100-nt sequencing was performed on an Illumina HiSeq2000 or HiSeq1500 platform (Illumina, San Diego, CA, USA). Fast read files were generated from the sequencing platform via the Illumina pipeline. Adapter sequences in the raw data were removed and low quality reads with low base quality were discarded. Paired-end reads from FASTQ files were aligned to the human reference genome hs38DH using the Burrows-Wheeler Alignment (BWA) package MEM algorithm. Resulting Binary Alignment Map (BAM) files were post-processed with Sentieon DNAseq software (Sentieon, Inc., San Jose, CA, USA), implementing the GATK 3.5 Best Practices protocol, and variant call format (VCF) files were generated. The obtained 15 Gb of aligned sequence data resulted in approximately 75× the median coverage of the target capture regions with 97.5% of target bases covered at least 10×. Capture performance statistics were calculated using CollectHsMetrics in Picard 2.17.10. The alignments were viewed with the Integrative Genomics Viewer [8]. SNVs (single-nucleotide variants) and indels (small insertion/deletion) variants were called using the GATK Unified Genotyper. Annovar was used for initial variant annotation [9].

### 2.5. Bioinformatic Analyses

Exome sequencing identified on average 156,000 SNVs and 29,000 indels per exome, of which 89,000 and 17,000, respectively, were intergenic or intronic (but not affecting splice sites) and removed from further consideration. Only variants with an impact on protein secondary structure (missense, nonsense, and frameshift) and on splicing were retained. Further filtering was based on Phred quality scores, minor allele frequency in the ExAC (Exome Aggregation Consortium) and gnomAD (The Genome Aggregation Database) databases (below 1% were retained), and association with human phenotype ontology (HPO) terms [10]. The HPO term used was HP:0011805 Abnormal muscle morphology. Prioritization was based on the predicted effect on the protein, predicted pathogenicity (assessed with Mutation Taster, Mutation Assessor, PolyPhen2, Provean, CADD, and SIFT), and known association with myopathic phenotypes. The variants on the final list were individually assessed together by both geneticists and clinicians according to the guidelines of the American College of Medical Genetics and Genomics [11], with emphasis on the possible match with the actual phenotype of each patient.

Selected variants (including all putative causative mutations and discussed phenotype variants) were confirmed with direct fluorescence-based sequencing (ABI 3130 Genetic Analyzer, Applied Biosystems, Waltham, MA, USA).

## 3. Results

### 3.1. Whole Exome Sequencing 

Using exome sequencing, we identified putative pathogenic mutations in known myopathy-related genes in eight of the eleven MFM cases (73%). These were pathogenic or likely pathogenic according to the American College of Medical Genetics and Genomics (ACMG) criteria and matched the respective patient’s phenotype (see Table 2).

Dominant mutations were identified in *DES* (two cases), *CRYAB* (one case), and *TPM3* (one case) and found to segregate with the phenotype in a family or to occur de novo (patient 4). Five further patients (5-8b) had mutations in both copies of the *SELENON* gene, and family analysis confirmed that they were inherited from both parents (biparental inheritance).

Filtering of the variants annotated based on the HPO terms associated with genes returned between 32 and 64 variants per genome (47.3 on average) that could be related to muscle abnormality. These variants were found in various myopathy-associated genes, in genes associated with myofibrillar myopathy, limb-girdle muscular dystrophy, rigid spine congenital muscular dystrophy, nemaline myopathy, multiminicore myopathy, collagen myopathy, tubular myopathy, or Ehlers-Danlos syndrome. Full results including details of all the variants identified after filtering for each patient and results of the pathway mapping analysis are provided in a supplementary table (see Supplementary Table S1).

### 3.2. Clinical Data

The clinical data of the patients are shown in Table 3.

**Figure 1:**
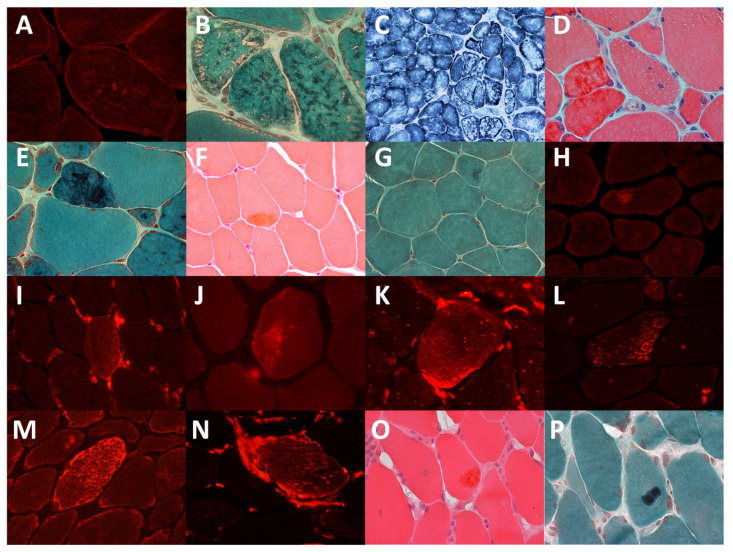
Immunohistochemistry and light microscope images of muscles of the patients. (**A**). Patient 2: desmin aggregates (**B**). Patient 2: small reddish or dark inclusions in the Gomori trichrome staining. (**C**). Patient 2: lobulated fibers visible in the succinate dehydrogenase staining. (**D**). Patient 3: dark red material in the hematoxylin and eosin staining. (**E**). Patient 3: dark blue material in the trichrome staining. (**F**). Patient 5: acidophilic inclusions in the hematoxylin and eosin staining, (**G**) Patient 5: acidophilic inclusions in the trichrome staining. (**H**). Patient 5: desmin aggregates. (**I**). Patient 5: vimentin aggregates. (**J**). Patient 6: CRYAB aggregates. (**K**). Patient 6: vimentin aggregates. (**L**). Patient 11: CRYAB aggregates. (**M**). Patient 11: desmin aggregates. (**N**). Patient 11: vimentin aggregates. (**O**). Patient 11: large acidophilic inclusions in the hematoxylin and eosin staining, (**P**). Patient 11: acidophilic inclusions in the trichrome staining.

Histological analysis documented myopathic features in all cases (Selected specimens—Figure 1).

**Figure 2:**
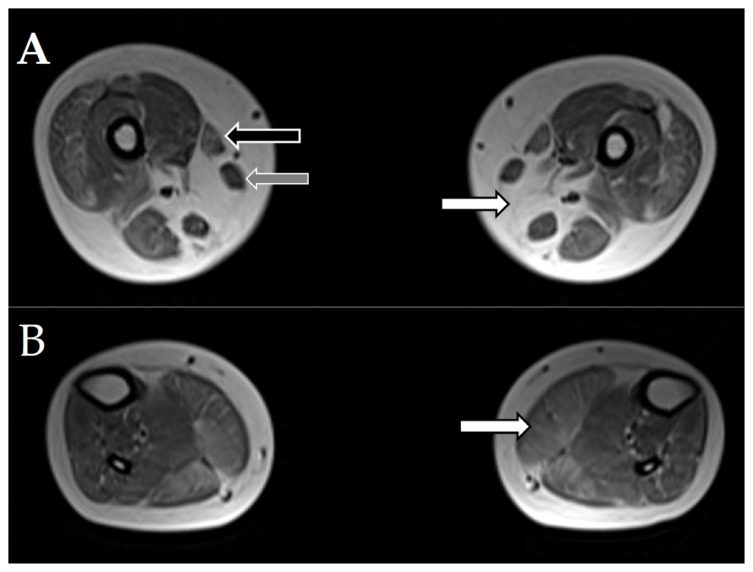
Magnetic resonance imaging (MRI) of muscles of patients 7 and 8a with *SELENON* mutations. Selected axial T1W images of the muscles. (**A**). White arrow points to the absence of the semimembranosus muscle. Black arrow points to the moderately affected sartorius muscle. Grey arrow points to the intact gracilis muscle. (**B**). White arrow points to moderate, diffuse changes in the gastrocnemius muscle.

In addition, MRI was performed for two patients (7, 8a) with *SELENON* mutations (Figure 2). It showed an absence of the semimembranosus muscle, characteristic of *SELENON*-related cases, moderate-to-severe changes in the sartorius muscle, and sparing of the gracilis muscle.

**Figure 3:**
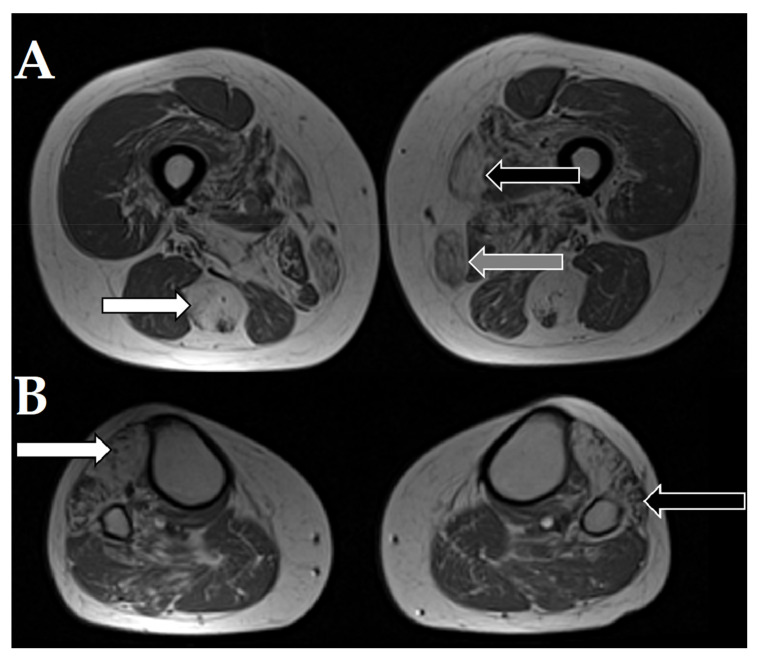
Magnetic resonance imaging of selected axial T1W images of muscles of the patient 1a with desminopathy. (**A**). White arrow points to severe fatty atrophy in the semitendinosus muscle. Black arrow points to the sartorius muscle. Grey arrow points to the gracilis muscle. (**B**). White arrow points to severe changes in the tibialis anterior muscle. Black arrow points to the affected peroneus muscles.

The MRI of the patient 1a with desminopathy (previously reported by Fichna et al., 2014 [5]) showed characteristic severe changes in the semitendinosus muscle and moderate-to-severe changes in the adductor magnus, vastus intermedius, and gracilis muscles with relative sparing of the rectus femoris, vastus lateralis, adductor longus, and the long head of biceps femoris (Figure 3).

## 4. Discussion

We completed and reported on the first exome-wide analysis in a group of MFM patients and their families from the Polish population. As a result of bioinformatic filtering we found, on average, 47.4 rare variants per patient in myopathy-related genes. These genes were already associated with diverse diseases of the skeletal muscle, but not necessarily with MFM.

Among the identified variants, causative variants were identified in eight out of eleven cases (73%). They were pathogenic according to the ACMG criteria and are consistent with the clinical characteristics of the patients. 

In cases with typical dominant inheritance, the identified causative mutations were mostly in the known MFM-related genes (*DES* and *CRYAB*), but in one dominant case, a known causative mutation in *TPM3*, not commonly associated with MFM, was found. Variants in the *TPM3* gene encoding γ-tropomyosin expressed in type 1 slow muscle fibers have been mostly reported with three other types of muscle disease: cap myopathy, congenital myopathy with fiber type disproportion, and nemaline myopathy.

Interestingly, the apparently sporadic cases appeared to be recessive rather than de novo dominant forms of MFM. In three such cases, causative mutations were found in the *SELENON* gene commonly associated with rigid spine syndrome but also with congenital myopathy with fiber type disproportion. However, some *SELENON* mutations have also been reported in MFM-like cases [12] and were considered in similar phenotypes [13]. All three variants identified in the present study have already been reported, with p.Arg466Gln being one of the first *SELENON* mutations associated with a muscle disease [14]. The c.713dupA variant most common in our cohort has been reported at least five times, most recently in three cases in the Czech population [15]. The same study also reported three cases of the c.997_1000delGTGC mutation found by us in a single patient. The distribution of MFM subtypes suggests *SELENON* cases to be fairly common in the Polish and Czech populations. The gene should be of special interest in cases with a childhood onset, rigid spine phenotype, and/or floppy baby syndrome. In addition, an MRI scan indicating an absence of the semimembranosus muscle and sparing of the gracilis muscle is strongly indicative of *SELENON*-related cases (Figure 1). Magnetic resonance imaging is increasingly being used to characterize the severity and pattern of individual muscle involvement. It could provide a very useful noninvasive diagnostic tool for the detection and quantification of myopathic changes during the clinical workup.

In the three unsolved cases of our study, the pathogenic mutations could likely be located in noncoding (regulatory or deep intronic) regions. Copy number variation would be missed by exome sequencing, but could influence the phenotype. Moreover, in sporadic or first cases in a family, a post-zygotic mutation affecting muscle but not the blood cells could cause the disease.

Additional variants that at the present state of knowledge could not be identified as causative still could explain both the phenotypic overlap, and the inter- and intrafamilial differences.

In a few cases, the patient’s clinical phenotype could be plausibly explained by mutations in more than one gene. Patient no. 2 had a much earlier onset and a more severe disease course than his also-affected mother. They both have a p.Gln348Pro causal missense mutation in the *DES* gene encoding desmin. Mutations in *DES* are common cause of MFM, especially with cardiac involvement [16], however, the son additionally carries a private *DNAJB6* c.962C>T (p.Ser321Leu) mutation, which could explain his more severe course of the disease. The ACMG criteria classify the variant as likely benign, mainly due to its common frequency in the general population (gnomAd MAF = 0.0004437). Verdicts of the prediction programs are inconclusive (SIFT—deleterious, Polyphen, MutationAssessor and MutationTaster—benign). In addition, most of the known pathogenic mutations in *DNAJB6* were reported in exon 5, not exon 10, and affect the G/F domain characteristic for the DNAJA and DNAJB proteins. On the other hand, missense mutations in *DNAJB6* are rarely benign but are a common cause of myofibrillar disintegration. The DNAJB6 protein is localized primarily at the Z-disk where it is involved in the maintenance of keratin filament organization [17]. Keratins 18 and 19 seem to play a role complementary to desmin in assembling intermediate filament networks and transducing the contractile force [18]. Even if the identified *DNAJB6* variant is not pathogenic in itself, it could be responsible for the earlier and more severe progression of the desmin-related disease.

In family 1, with a clearly causative *DES* mutation, a slightly more severe disease course of patient 1a compared to her cousin, patient 1b, could be an effect of phenotype-affecting variants. Mutations in the RYR1 gene have been reported in mild clinical forms of rigid spine syndrome [19] and in cases similar to myofibrillar myopathy [20]. Possibly the novel variant NM_000540.3:c.12809C>A in this gene identified in patient 1a could be responsible for the difference in the clinical manifestation between patients from the same family. 

The *TPM3* p.Arg168Cys variant identified in patient no. 4 was reported several times in patients presenting a full spectrum of diseases associated with *TPM3* mutations [21,22,23]. In addition, other mutations in the same codon, p.Arg168Gly and p.Arg168Lys, have been reported. Although some TPM3 variants are associated with a specific form of myopathy [24], the fiber-type distribution pattern and the pattern of protein inclusions often vary widely, even among patients with the same *TPM3* mutation. Therefore, it is likely that the phenotype is modified by other genomic variants. In the case of patient no. 4, the *LDB3*(NM_007078.3):c.1300G>T (p.Ala434Ser) variant seems particularly interesting as it affects a gene already associated with MFM. The ACMG criteria suggest the variant is likely benign as out of 38 missense *LDB3* variants of unknown significance, 35 appeared to be benign, and most of the pathogenic mutations reported were in exon 6, not in exon 10. However, the prediction programs do not agree on the variant pathogenicity (SIFT, Polyphen—benign, MutationAssessor and MutationTaster—damaging). In addition, the variant is not reported in the gnomAd database. Nevertheless, even if not pathogenic by itself, this variant could affect the phenotype of a patient carrying a causative mutation in another gene.

In other cases, the copious variants found in many muscle-related genes seem likely to modify or exacerbate phenotypic manifestation of the major pathogenic mutations, however, selecting co-causative variants from among those classified as potentially modifying was not possible. Discrimination between genuine phenotype-affecting variants and the thousands of insignificant variants harbored by each individual is one of the most challenging tasks posed by the introduction of genomic-scale analyses. Indeed, in all the cases studied here we encountered novel rare variants that could be related to myopathies. Some of these genes could be grouped together with known MFM-associated genes according to the structural or functional association of their products. However, their relevance could not be established based on the inheritance mode, patient’s phenotype, and known effects of other mutations in these genes.

Numerous muscular disorders overlap phenotypically with MFM. NGS-based genetic analyses can resolve clinical dilemmas and facilitate exact diagnosis [25,26]. Additionally, with the reporting of new cases, the reported clinical manifestations of mutations in a given gene are likely to expand. Recent large sequencing studies show that the genetic background of muscle diseases is more complex than previously appreciated [27,28,29]. In addition, our data suggest that mutations in more than one gene in a patient can result in a more severe phenotype. The observed phenotypic variability within a given MFM subtype, particularly between patients with the same causative mutation, indicate a strong influence of disease-modifying genes. Indeed, a common polymorphism in the *TIA1* gene has been shown to be a necessary factor in *SQSTM*-related MFM [30]. Digenic inheritance has been proven in other muscular disorders, such as a subtype of facioscapulohumeral muscular dystrophy [31,32] and congenital myasthenic syndrome [33]. Unequivocal identification of risk, phenotype-modifying or co-causative factors, is a major challenge that requires comprehensive bioinformatic analysis of combined genomic and clinical data followed by functional in vitro studies [34].

Myofibril proteins form a complex machinery whose functional or structural impairment can lead to progressive dysfunction of the muscle. The number of rare variants identified in genes already associated with muscle diseases was higher than in patients with non-muscular diseases (i.e., neurodevelopmental) studied by our group. While it suggests their aggregate influence on the phenotype, this conclusion must be verified on a larger cohort of both patients and controls. The variant burden in many genes associated with muscular diseases must not be overlooked, as accumulation of minor changes, even those without a sufficient influence when present in isolation, could result in dramatic phenotype changes. Such oligogenic or even polygenic etiology seems most likely in unsolved cases, where the main causative mutation cannot be identified. On the other hand, some of the mutations deemed disease-causing prior to the advent of NGS might turn out to have a modifying effect only or incomplete penetrance and require additional variants in other genes to result in pathology.

## 5. Conclusions

The widespread application of next generation sequencing in studies on the genetic basis of various diseases, including muscle disorders, has brought ample data challenging the classical “one gene—one phenotype” paradigm [35] and underscores the heterogeneity and complexity of the genome [36,37].

Our results emphasize the continuum of genetic causality as they show that mutations in many genes can result in MFM-like diseases. The causative mutations found in *TPM3* and *SELENON*—genes associated not only with MFM—highlight the phenotypic heterogeneity and clinical overlap between muscular disorders. Mutations in genes originally identified in patients with other muscle diseases, such as cap myopathy or minicore syndromes, produce a wide spectrum of phenotypes.

Although it is frequently not possible to evaluate the weight of additional putative phenotype-affecting mutations, comprehensive analysis of variants suggests that cumulative “variant load” may likely contribute to the observed phenotypic variability. This could explain the difference in clinical manifestation among cases with the same causative mutations, sometimes even between related patients. Even though the majority of the MFM cases presented here could be explained by mutations in a single gene, the identification of numerous variants in genes associated with various myopathies, some of which could even be related to specific symptoms of the patients, is intriguing. The oligogenic background of MFM is visible also in some of the patients harboring a clearly pathogenic mutation.

## Figures and Tables

**Table 1:** Red flags characteristic for myofibrillar myopathies (MFM) and their frequency in patients. CK: creatine kinase.

Features	Frequency (and Patient Numbers)
Family history positive	7/13 (patients no 1a, 1b, 2, 3, 8a, 8b, 11
Predominant distal weakness at the onset (distal myopathy) or Equivocal distal and proximal weakness of the lower limbs	5/13 (1a, 2, 3, 6, 9) 7/13(1b, 4, 5, 7, 8b, 10, 11)
Rigid spine	7/13 (4, 5, 6, 7, 9, 10,11)
Cataract	2/11 (3, 9)
Cardiac involvement	4/13 (2, 3, 6, 10)
Early respiratory involvement	9/13 (4, 5, 6, 7, 8a, 8b, 9, 10, 11)
Normal or moderate elevated CK (maximum 2 x normal limit)	All patients
Myopathic changes in electromyography (EMG)	12/13 (1a, 1b, 2, 3, 4, 6, 7, 8a, 8b, 9, 10, 11)
Changes in muscle biopsy suggestive of MFM	9/13 (1b, 2, 3, 4, 5, 6, 7, 9, 10)

**Table 2:** Putative causative mutations and genes with potentially phenotype-influencing variants in MFM patients.

Patient No.	Onset	Inheritance	Causal Genotype	ACMG Criteria Met	Additional Variants in Following Genes
**1a**	adulthood	dominant	*DES* c.1069_1077del (p.Ala357_Glu359del)	PM1, PM2, PM4, PP3, PP5	*NEB*, *RYR1*, *MYH3*, *COL6A3*, *HSPG2*, *FLNC*, *RBM20*, *TTN*
**1b**	adulthood	dominant	*DES* c.1069_1077del (p.Ala357_Glu359del)	PM1, PM2, PM4, PP3, PP5	*FKRP*, *COL6A3*, *ITGA7*, *HSPG2*, *CHKB*, *FLNC*, *RBM20*, *TTN*
**2**	early adulthood	dominant	*DES* c.1043A>C (p.Gln348Pro)	PM1, PM2, PP2, PP3, PP5	*DNAJB6*, *DYSF*, *SYNE1*, *HSPG2*, *FLNC*, *TTN*
**3**	early adulthood	dominant	*CRYAB* c.326A>C (p.Asp109Ala)	PM1, PM2, PM5, PP2, PP3	*SYNE1*, *HSPG2*, *TTN*
**4**	childhood	sporadic	*TPM3* c.502C>T (p.Arg168Cys)	PM1, PM2, PM5, PP2, PP3, PP5	*LDB3*, *GCH1*, *POMGNT2*, *ACTN3*
**5**	childhood	sporadic	*SELENON* c.713dupA (p.Asn238LysfsTer63) c.713dupA (p.Asn238LysfsTer63)	PVS1, PM2, PP3, PP5	*NEB*, *POMGNT2*, *DMD*, *TMPO*, *SYNE1*, *SYNE2*
**6**	early adulthood	sporadic	*SELENON* c.997_1000del (Val333Profs) c.1397G>A (p.Arg466Gln)	PVS1, PM2, PP3, PP5 PM2, PP2, PP3, PP5	*FLNC*, *COL6A2*, *KBTBD13*, *SYNE2*, *POMGNT2*, *SCN4A*, *TTN*, *SACS*
**7**	childhood	sporadic	*SELENON* c.713dupA (p.Asn238LysfsTer63) c.713dupA (p.Asn238LysfsTer63)	PVS1, PM2, PP3, PP5	*TTN*, *ITGA7*, *POMT2*, *HSPG2*, *LAMA2*, *LDB3*, *SYNE1*, *SYNE2*
**8a**	childhood	recessive	*SELENON* c.713dupA (p.Asn238LysfsTer63) c.997_1000del (Val333Profs)	PVS1, PM2, PP3, PP5 PVS1, PM2, PP3, PP5	*ACTN2*, *NEB*, *TTN*, *SQSTM1*, *SYNE1*, *ANO5*, *ITGA7*, *SCN4A*
**8b**	childhood	recessive	*SELENON* c.713dupA (p.Asn238LysfsTer63) c.997_1000del (Val333Profs)	PVS1, PM2, PP3, PP5 PVS1, PM2, PP3, PP5	
**9**	childhood	sporadic	-		*DYSF*, *LAMA2*, *NEB*, *PHKB*, *PUS1*, *TTN*
**10**	childhood	sporadic	-		*NEB*, *COL6A3*, *DSP*, *SACS*, *TTN*
**11**	childhood	sporadic	-		*LAMA4*, *GLA*, *TTN*, *FKBP14*, *SLC22A5*

RefSeq transcript reference sequences as in the LOVD database: DES—NM_001927.3, DNAJB6—NM_058246.3, CRYAB—NM_001885.1, TPM3—NM_152263.2, SELENON—NM_020451.2, ACMG—American College of Medical Genetics and Genomics.

**Table 3:** Clinical features of the patients.

No.	Family History/Gene	Sex	Age at Onset	First Symptoms	Neurological Examination	Ability to Walk	CK (U/L) 26-192	EMG	Cardiac/Pulmonary Involvement
**1a**	positive *DES*	F	46	Gait disorders, dyspnea after physical effort	Moderate weakness: proximal < distal, upper < lower; wasting of the lower limb muscles; deep tendon reflexes diminished in the lower limbs	Unable to go on the heels and on the toes.	333	Myopathic	None
	Muscle MRI: severe symmetric changes in the iliopsoas, semitendinosus, and tibialis anterior muscles and slightly less pronounced abnormalities in the sartorius, gracilis, adductor magnus, short head of the biceps femoris, vastus, and gluteus muscles.								
**1b**	positive *DES*	F	39	Gait disorders	Mild weakness: proximal = distal, upper = lower	Normal	192	Myopathic	None
	Muscle biopsy: Biceps brachii LM: H&E staining revealed that the fascicular architecture of the muscle showed marked variability in fiber size and shape, with atrophy of the fibers in the form of “nuclear clumps”, several fibers with multiple centrally located nuclei. Trichrome staining revealed reddish inclusions in a few fibers. EM: Disintegration of myofibrils and accumulation of degradation products and desmin inclusions.								
**2**	positive *DES*	M	21	Fasciculations, muscle spasms, dropping feet	High arched palate; atrophy of the lower limb muscles; moderate weakness: proximal < distal, upper < lower,	Not on heels	191	Myopathic	Incomplete right bundle branch block (RBBB)
	Muscle biopsy: Quadriceps femoris (vastus lateralis) muscle LM: H&E staining revealed atrophy of the fibers partially preserved as “nuclear clumps”. Trichrome staining revealed in some fibers small reddish or dark inclusions. Oxidative enzymes staining revealed marked atrophy of type 2 fibers, numerous lobulated fibers. EM: disintegration of myofibrils and accumulation of degradation products into inclusions containing desmin. IHC: Desmin aggregates.								
**3**	positive *CRYAB*	F	26	Progressive gait disorder, dropping feet	Moderate weakness: proximal < distal, upper < lower, deep tendon reflexes diminished in the lower limbs; dysphagia, dysphonia Cataracts surgery at the age of 35 y	Lost at the age of 58	250	Myopathic	Cardiomyopathy at the age of 56. Respiratory failure at the age of 56.Noninvasive ventilation (BiPAP) required during the night.
	Muscle biopsy: Quadriceps femoris (vastus lateralis) LM: H&E staining revealed moderate myopathic changes, variability in fiber size and shape, atrophy of fibers, and some fibers preserved in the form of “nuclear clumps”. The presence of dark red material in (HE) staining and dark blue material in trichrome; lobulated fibers in succinate dehydrogenase (SDH) staining. EM: disorganization of the sarcomeres, presence of electron dense material. IHC: CRYAB aggregates located beneath the sarcolemma and within the fibers, both in atrophic and normal-sized ones.								
**4**	Sporadic *TPM3*	F	9	Progressive decline in physical activity, scoliosis	Rigid spine, high arched palate, mild weakness: proximal = distal, upper < lower; dysmorphic long myopathic face; ankle joint contractures	Not on heels	44	Myopathic	On plethysmography, slight restriction, not ventilated.
	Muscle biopsy: Biceps brachii LM: Small hypoplastic type 1 muscle fibers, normal type 2 fibers.								
**5**	sporadic *SELENON*	M	4	Progressive gait disorder, motor development delay	Rigid spine dysmorphic myopathic face, clinodactyly, mild weakness: proximal = distal, upper < lower	Normal	126	Normal	On plethysmography, restriction.
	Muscle biopsy: Quadriceps femoris (vastus lateralis) LM: H&E staining revealed muscle fibers with different diameters (normal, atrophic, and hypertrophic); in some fibers there were acidophilic inclusions in the cytoplasm, which in trichrome staining turned blue. IHC: Desmin and vimentin aggregates.								
**6**	sporadic *SELENON*	F	7	Rigid spine, scoliosis, fatigue	Moderate weakness: proximal < distal, upper < lower, deep tendon reflexes diminished in the lower limbs; weakness of the axial muscles, hyperlordosis, ankle joint contractures	Not on heels	486	Myopathic	On plethysmography, restriction, not ventilated; decreased left ventricular contractility.
	Muscle biopsy: Quadriceps femoris (vastus lateralis) biopsy at the age of 8—discrete myopathic changes. IHC: Lamin A/C, lamin B2, and emerin aggregates. At the age of 17: LM: H&E staining revealed variability in fiber size and shape, some atrophied fibers preserved as “nuclear clumps”, necrosis in a few fibers, in trichrome staining, inclusions of dark red material. Oxidative enzyme stainings showed lobulated fibers. IHC: CRYAB and vimentin aggregates.								
**7**	sporadic *SELENON*	M	2	floppy baby syndrome, motor development delay, scoliosis,	Mild weakness: proximal = distal, upper < lower rigid spine, dysmorphic myopathic face, high arched palate, chest deformation,	Waddling gait	540	Myopathic	Chronic respiratory failure at age 4 noninvasive ventilation (BiPAP) required during the night.
	Muscle MRI: absence of the semimembranosus muscle, severe fatty infiltration in the cervical and thoracic paraspinal, serratus anterior, and gluteus maximus muscles, moderate changes in the adductor magnus, sartorius, vastus lateralis, and gastrocnemius muscles and sternocleidomastoid muscle atrophy								
**8a**	recessive *SELENON*	M	3 mths	floppy baby syndrome, motor development delay, dysphagia	Moderate weakness: proximal > distal, upper < lower rigid spine, dysmorphic myopathic face, high arched palate, wadding gait	Not on heels	114	Myopathic	Chronic respiratory failure after pneumonia at age 4 noninvasive ventilation (BiPAP) required during the night.
	Muscle MRI: absence of the semimembranosus muscle, severe fatty infiltration in the cervical paraspinal, serratus anterior, gluteus maximus, and sartorius muscles, moderate changes in the thoracic paraspinal, latissimus dorsi, pectoralis major, rotator cuff, adductor magnus, vastus lateralis, biceps femoris, semitendinosus, and gastrocnemius muscles and SCM atrophy								
**8b**	recessive *SEPN1*	F	At birth	Significant dysphagia, silent crying, floppy baby syndrome, motor development delay,	Mild weakness: proximal = distal, upper < lower, dysmorphic myopathic face, high arched palate, wadding gait,	Not on heels	150	Myopathic	Chronic respiratory failure after at age 3 noninvasive ventilation (BiPAP) required during the night.
	Muscle biopsy/Muscle MRI: Not performed								
**9**	sporadic (not found)	M		Motor development delay, congenital defect of fingers and toes, epilepsy	Severe muscle weakness: proximal < distal, upper < lower, rigid spine, dysmorphic myopathic face, right ptosis, high arched palate, bilateral cataract, short stature	Not on heels	274	Myopathic	On plethysmography, restriction at age 10, noninvasive ventilation (BiPAP) required during the night.
	Muscle biopsy: Quadriceps muscle (vastus lateralis) LM: H&E staining revealed muscle fibers with different diameters (normal and hypertrophic), in some fibers acidophilic inclusions are visible in the cytoplasm, which in trichrome staining turned pink. IHC: CRYAB in aggregate-like structures.								
**10**	sporadic (not found)	M	2	Progressive gait disorders, fatigue, thin bones building, flaccid muscles	Mild weakness: proximal = distal, upper < lower rigid spine	Normal	54	Myopathic	Slightly decreased vital capacity at spirometry supraventricular wandering pacemaker.
	Muscle biopsy: Biceps brachii LM: Discrete unspecific myopathic changes with multifocal defect of enzymatic activity in type 1 muscle fibers. IHC: Lamin A/C, laminB2 and emerin aggregates.								
**11**	positive (not found)	M	7	Gait disorders, scoliosis	Mild weaknes sproximal = distal, Upper < lower, deep tendon reflexes diminished in the lower limbs; rigid spine	Normal	204	Myopathic	On plethysmography-restriction
	Muscle biopsy: Quadriceps femoris (vastus lateralis) LM: H&E staining revealed muscle fibers with different diameters (normal and atrophic), several fibers with necrosis and phagocytosis, large acidophilic inclusions are visible in the cytoplasm, which in trichrome staining turned dark blue. IHC: Desmin, CRYAB, and vimentin aggregates. EM: Disintegration of myofibrils, accumulation of mitochondria with irregular cristae and numerous lipid droplets.								

LM—light microscope; EM—electron microscope; IHC—immunohistochemistry, RBBB—right bundle branch block, BiPAP—bilevel positive airway pressure.

## Data Availability

All data generated during this study are included in the supplementary file. The raw data analyzed during the current study are available from the corresponding author on reasonable request.

## Supplementary Materials

The following is available online at https://www.mdpi.com/2077-0383/10/5/914/s1, Table S1: Rare variants in genes associated with myopathies in MFM patients.
